# Thermodynamics Analysis and Removal of P in a P-(M)-H_2_O System

**DOI:** 10.3390/molecules26113342

**Published:** 2021-06-02

**Authors:** Hao Peng, Jing Guo, Hongzhi Qiu, Caiqiong Wang, Chenyu Zhang, Zhihui Hao, Yating Rao, Yanhong Gong

**Affiliations:** Chongqing Key Laboratory of Inorganic Special Functional Materials, College of Chemistry and Chemical Engineering, Yangtze Normal University, Fuling, Chongqing 408100, China; 20171110@yznu.edu.cn (J.G.); q780173781@126.com (H.Q.); wcq18290390314@163.com (C.W.); hzh3229224722@126.com (C.Z.); zcy20010126@126.com (Z.H.); rytlill@163.com (Y.R.); scarlett20001212@163.com (Y.G.)

**Keywords:** thermodynamics, removal, phosphorus

## Abstract

In order to efficiently remove phosphorus, thermodynamic equilibrium diagrams of the P-H_2_O system and P-M-H_2_O system (M stands for Fe, Al, Ca, Mg) were analyzed by software from Visual MINTEQ to identify the existence of phosphorus ions and metal ions as pH ranged from 1 to 14. The results showed that the phosphorus ions existed in the form of H_3_PO_4_, H_2_PO_4_^−^, HPO_4_^2−^, and PO_4_^3−^. Among them, H_2_PO_4_^−^ and HPO_4_^2−^ were the main species in the acidic medium (99% at pH = 5) and alkaline medium (97.9% at pH = 10). In the P-Fe-H_2_O system ((P) = 0.01 mol/L, (Fe^3+^) = 0.01 mol/L), H_2_PO_4_^−^ was transformed to FeHPO_4_^+^ at pH = 0–7 due to the existence of Fe^3+^ and then transformed to HPO_4_^2−^ at pH > 6 as the Fe^3+^ was mostly precipitated. In the P-Ca-H_2_O system ((P) = 0.01 mol/L, (Ca^2+^) = 0.015 mol/L), the main species in the acidic medium was CaH_2_PO_4_^+^ and HPO_4_^2−^, and then transformed to CaPO_4_^−^ at pH > 7. In the P-Mg-H_2_O system ((P) = 0.01 mol/L, (Mg^2+^) = 0.015 mol/L), the main species in the acidic medium was H_2_PO_4_^−^ and then transformed to MgHPO_4_ at pH = 5–10, and finally transformed to MgPO_4_^−^ as pH increased. The verification experiments (precipitation experiments) with single metal ions confirmed that the theoretical analysis could be used to guide the actual experiments.

## 1. Introduction

Phosphorus plays an important role in living organisms as it is one of the main components of cell structure [1]. Usually, phosphorus exists in three species (orthophosphate, polyphosphate, and organic phosphorus) in solution. Furthermore, the primary phosphorus compounds are generally orthophosphates [2]. The overuse and inefficient use of phosphorus lead to the eutrophication problem in natural water, which is a serious global problem that needs to be treated [3,4].

Phosphorus mitigation is expensive and difficult in the waste stream. Many methods have been developed for phosphorus removal. The conventional biological methods have difficulty in removing phosphorus due to the inherent limitations of the activated sludge method. The removal efficiency of phosphorus is less than 30%, and the residual phosphorus concentration is still over the wastewater discharge guideline [5]. As a result of catastrophic environmental implications, including eutrophication and red tide, the governments have established some biological wastewater treatment systems to treat the wastewater containing phosphorus and limited the emission standard for residual phosphorus concentration (0.5–1 mg TP/L in the USA, <0.2 mg TP/L in South Korea, and 1–2 mg TP/L in France) [6]. The conventional biological treatment processes can only achieve low removal efficiency of phosphorus due to large volume requirements and long hydraulic retention time. Thus, the development of advanced technology to improve phosphorus removal efficiency is needed, including easy installation, short intention time, little space, low operation cost, and low capital investment [2,7,8,9,10]. Enhanced biological phosphorus removal processes have attracted much more attention [11,12,13,14,15]. The key factor in the enhanced biological phosphorus removal process is the use of phosphorus accumulating organisms. Under anaerobic conditions, the stored poly-phosphate (poly-P) is hydrolyzed to supply energy for the polyhydroxyalkanoates uptake. During the subsequent aerobic process, phosphorus accumulating organisms absorb the excessive amounts of phosphorus for poly-P. Finally, the phosphorus is removed when the waste-activated sludge is separated from the treated wastewater at the end of the aerobic stage. In the whole process, stable phosphorus removal efficiency is hard to maintain. Thus, some physicochemical treatments have come to the forefront [16,17,18]. Among them, chemical precipitation methods are widely used for phosphorus removal with metal ion salts. Calcium salts, such as CaCl_2_, Ca (NO_3_)_2_, are often added for the removal of phosphorus as calcium and phosphorus have a strong affinity and could form insoluble Ca_3_(PO_4_)_2_ [19]. The concentration of phosphorus can be reduced from 15.1 ppmw to 0.2 ppmw with the addition of magnesium in acidic conditions [20]. Commonly, magnesium is used with ammonium for phosphorus removal called the magnesium ammonium phosphate method [21,22,23,24], but it is limited by co-precipitation, low separation efficiency caused by the separation equipment, serious ammonia wastewater, and ammonia gas pollution [25]. Ferric and aluminum are also used for phosphorus removal as they can generate co-precipitates with phosphate, and the hydroxyl complexes formed by hydrolyzing of Fe^3+^ and Al^3+^ show great adsorption performance for orthophosphate [26,27,28].

From the above analysis, the existing form of phosphorus and the metal ion in the solution has a great influence on phosphorus removal efficiency. Thus, the present work focuses on the existence of phosphorus ions and metal ions in solution at different pH ranges by employing the Visual MINTEQ software. This work aims to provide a theoretical basis for efficient phosphorus removal from wastewater.

## 2. Results

### 2.1. Thermodynamic Analysis of the P-H_2_O System

In the P-H_2_O system, the phosphorus existed in the form of HPO_4_^2−^, H_2_PO_4_^−^, H_3_PO_4_, and PO_4_^3−^. These four species were interchangeable with changed pH according to Equations (1)–(3).

(1) $$\left[PO_{4}{}^{3-}]{\left[H^{+}\right]}^{2}=10^{-17.650}[H_{2}PO_{4}{}^{-}\right]$$

(2) $$\left[HPO_{4}{}^{2-}][H^{+}]=10^{-6.418}[H_{2}PO_{4}{}^{-}\right]$$

(3) $$\;[H_{3}PO_{4}]=10^{1.772}[H^{+}][H_{2}PO_{4}{}^{-}]$$

**Figure 1:**
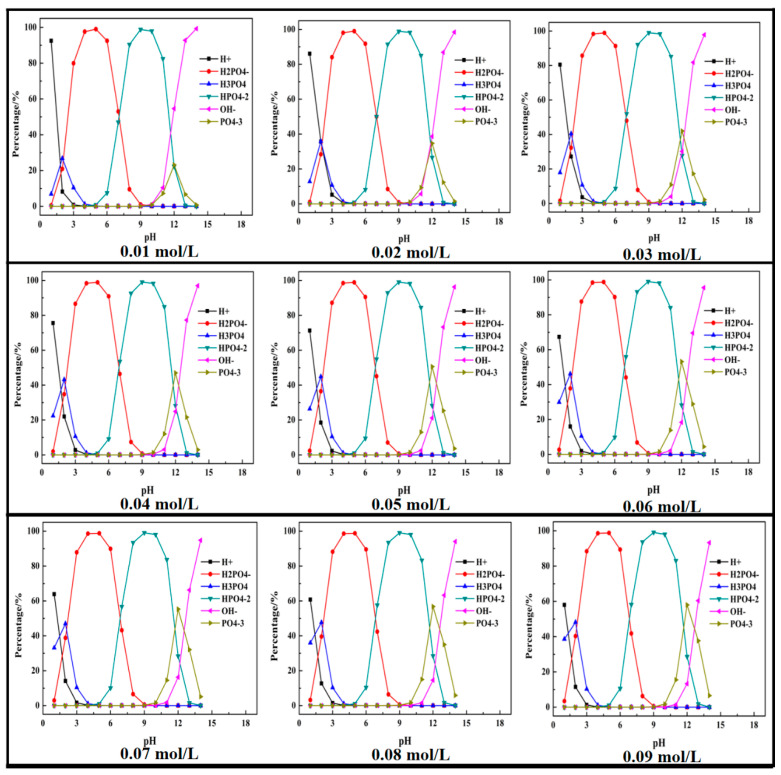
Mole fraction diagram of phosphorus species in the P-H_2_O system at 298 K at various pH ((P) = 0.01 to 0.90 mol/L).

Based on the above equations, thermodynamic studies of the P-H_2_O system were performed to determine the chemical state of ions in the wastewater. A mole fraction of different phosphorus species in the P-H_2_O system was plotted as the pH ranged from 1 to 14 and (P) = 0.01 mol/L to 0.09 mol/L. The results are shown in Figure 1.

It can be seen that the phosphorus mainly existed in the form of H_3_PO_4_, H_2_PO_4_^−^, HPO_4_^2−^, and PO_4_^3−^. With the increase in pH value, the phosphorus transformed to H_2_PO_4_^−^, HPO_4_^2−^ and PO_4_^3−^. The maximum percentage of H_3_PO_4_, H_2_PO_4_^−^, HPO_4_^2−^, PO_4_^3−^ was, respectively, 26.8% (at pH = 2), 99% (at pH = 5), 97.9% (when pH = 10), and 23.1% (at pH = 12). With the increase in phosphorus concentration, the percentage of H_2_PO_4_^−^ and HPO_4_^2−^ had no obvious change, while the maximum percentage of H_3_PO_4_ and PO_4_^3−^ increased with the increase in phosphorus concentration, but the pH was kept constant.

### 2.2. Thermodynamic Analysis of M-P-H_2_O System

#### 2.2.1. Fe (Ca, Mg)-P-H_2_O System

**Figure 2:**
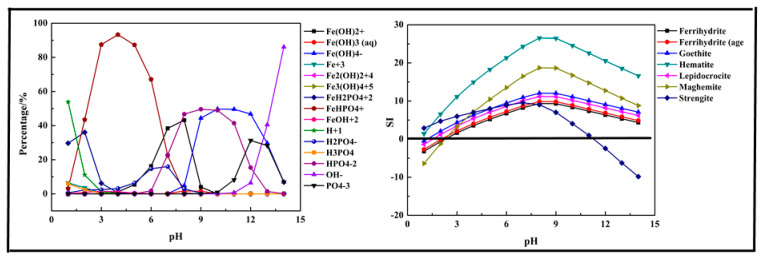
Percentage of species in the Fe-P-H_2_O system at 298 K at ranged pH ((P) = 0.01 mol/L, (Fe^3+^) = 0.01 mol/L).

**Figure 3:**
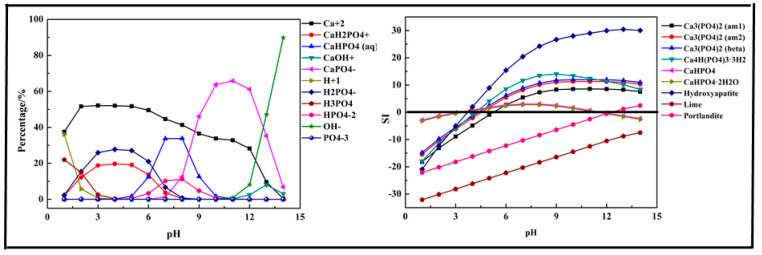
Percentage of species in the Ca-P-H_2_O system at 298 K at ranged pH ((P) = 0.01 mol/L, (Ca^2+^) = 0.015 mol/L).

**Figure 4:**
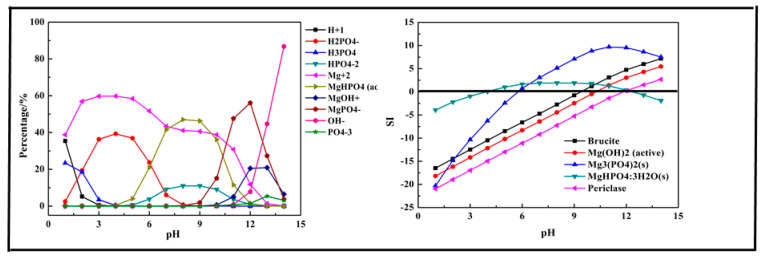
Percentage of species in the Mg-P-H_2_O system at 298 K at ranged pH ((P) = 0.01 mol/L, (Mg^2+^) = 0.015 mol/L).

In this section, a single metal salt was used to remove phosphorus from the wastewater, and Fe^3+^, Ca^2+^, and Mg^2+^ were selected. The composition in the phosphorus solution with single metal salt was simulated by Visual MINTEQ software. The results are shown in Figure 2, Figure 3 and Figure 4.

The results shown in Figure 2 indicated that the main species in the Fe-P-H_2_O system were Fe (OH)_2_^+^, Fe (OH)_3_ (aq), Fe (OH)_4_^−^, Fe^3+^, Fe_2_ (OH)_2_^4+^, Fe_3_ (OH)_4_^5+^, FeH_2_PO_4_^2+^, FeHPO_4_^+^, FeOH^2+^, H^+^, H_2_PO_4_^−^, H_3_PO_4_, HPO_4_^2−^, OH^−^, and PO_4_^3−^. Due to the existence of Fe^3+^, phosphorus first transformed into FeH_2_PO_4_^2+^ at pH < 2 and then transformed into FeHPO_4_^+^ at pH = 2–7. In an alkaline medium (pH > 7), the phosphorus and Fe^3+^ mainly existed in the form of HPO_4_^2−^ and Fe (OH)_4_^−^. The results shown in Figure 2 also display the predicted precipitation products. The main products of the reaction were Ferrihydrite (Fe_5_HO_8_·4H_2_O), Goethite (HFeO_2_), Hematite (Fe_2_O_3_), Lepidocrocite (FeO (OH)), Maghemite (γ-Fe_2_O_3_), and Strengite (FePO_4_·2H_2_O). Almost all the precipitation products were hydrolyzed Fe^3+^, but only Strengite is our aim. The suitable pH for precipitation of phosphorus with Fe^3+^ was below 11.

The results displayed in Figure 3 show that the main species in the Ca-P-H_2_O system were quite different from the Fe-P system. In the acidic medium (pH < 7), the phosphorus existed in the form of HPO_4_^2−^, H_2_PO_4_^−^. As pH value increased, it transformed into CaHPO_4_ (aq) and CaH_2_PO_4_^+^. When pH>7, it mainly existed in the form of CaPO_4_−. During the phosphorus removal process, there was no precipitation formed at pH < 4 because the SI of all predicted products was below 0. The first precipitation product, also the main product, was Hydroxyapatite (Ca_5_(PO_4_)_3_(OH)). As pH value increased, some other products might be generated, such as Ca_3_(PO_4_)_2_, Ca_4_H(PO_4_)_3_·3H_2_O, CaHPO_4_, and CaHPO_4_·2H_2_O. But CaHPO_4_ and CaHPO_4_·2H_2_O dissolved again at pH > 11. Above all, Ca^2+^ salts were used efficiently for phosphorus removal.

The results shown in Figure 4 indicate that the main species in the Mg-P-H_2_O system were H_2_PO_4_^−^, H_3_PO_4_, HPO_4_^2−^, Mg^2+^, MgHPO_4_ (aq), MgOH^+^, MgPO_4_^−^, OH^−^, and PO_4_^3−^. Phosphorus existing as H_3_PO_4_ first transformed into H_2_PO_4_^−^ at pH < 7 and then transformed into MgHPO_4_ (aq) and HPO_4_^2−^ at pH = 4–12, owing to the existence of Mg^2+^. As pH value increased to 9, MgPO_4_^−^ was the main form of phosphorus and up to 56.1% at pH = 12. The main products of the reaction were predicted as brucite (Mg(OH)_2_), Mg(OH)_2_, Mg_3_(PO_4_)_2_, MgHPO_4_·3H_2_O, and Periclase (MgO). The removal of phosphorus with Mg^2+^ should be conducted at pH > 6 and especially at pH > 10 as there was no precipitation generated at pH < 5 and only Mg_3_(PO_4_)_2,_ MgHPO_4_·3H_2_O generated after pH = 5. After pH = 11, almost all the predicted products generated except MgHPO_4_·3H_2_O, which dissolved again. The optimal pH ranged from 7 to 14.

In conclusion, the removal of phosphorus could achieve high efficiency in an alkaline medium. Using Fe^3+^ salts and Ca^2+^ salts more easily generated phosphorus precipitation and were more efficient in removing phosphorus.

#### 2.2.2. Two Salts System

**Figure 5:**
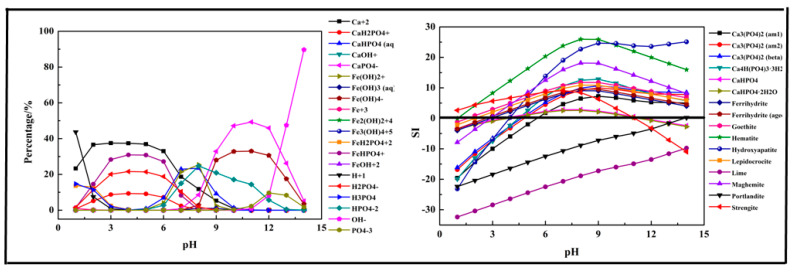
Percentage of species in the Fe-Ca-P-H_2_O system at 298 K at ranged pH ((P) = 0.01 mol/L, (Fe^3+^) = 0.005 mol/L, (Ca^2+^) = 0.0075 mol/L).

The results shown in Figure 5 indicate that the main species in the Fe-Ca-P-H_2_O system could be divided into three sections. At pH =1–6, CaH_2_PO_4_^+^, H_2_PO_4_^−^, FeHPO_4_^+^, and Ca^2+^ were the main species in the solution. As pH value increased, the above ions transformed into CaHPO_4_ (aq), HPO_4_^2−^, and Fe(OH)^2+^ at pH = 6–9. At pH > 9, the main species ions were CaPO_4_^−^, Fe (OH)_4_^−^, HPO_4_^2−^, OH^−^, and PO_4_^3−^. Strengite was the first predicted precipitation product generated in the precipitation process, and it dissolved again after pH > 11. Other precipitation products were generated after pH > 3, and the main phosphorus precipitation products were Hydroxyapatite, three kinds of Ca_3_(PO_4_)_2_ and Ca_4_H(PO_4_)_3_·3H_2_O. CaHPO_4,_ CaHPO_4_ generated at pH = 5–10 and then dissolved after pH > 11.

**Figure 6:**
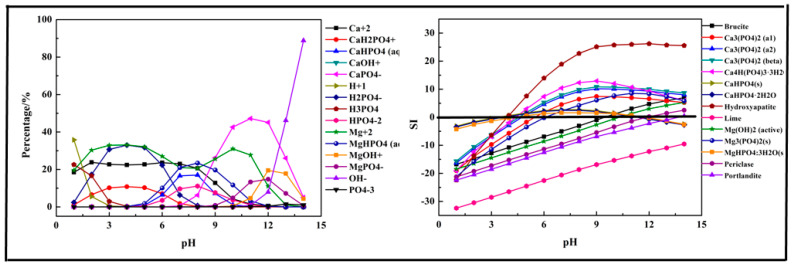
Percentage of species in the Ca-Mg-P-H_2_O system at 298 K at ranged pH ((P) = 0.01 mol/L, (Ca^2+^) = 0.0075 mol/L, (Mg^2+^) = 0.0075 mol/L).

The results shown in Figure 6 indicate that the main species in Ca-Mg-P-H_2_O system could be divided into three sections. At pH =1–6, H_2_PO_4_^−^, Ca^2+^, CaH_2_PO_4_^+^, and Mg^2+^ were the main species in the solution. As pH value increased, above ions transformed into CaHPO_4_ (aq), MgHPO_4_ (aq), HPO_4_^2−^ at pH = 6–9. At pH > 9, the main species ions were CaPO_4_^−^, MgOH^+^, and MgPO_4_^−^, Fe(OH)_4_^−^, OH^−^, and PO_4_^3−^. Hydroxyapatite (Ca_5_(PO_4_)_3_(OH)) was first precipitated at pH > 4, then Ca_4_H(PO_4_)_3_·3H_2_O, Ca_3_(PO_4_)_2_ followed. The precipitation containing Mg, such as Mg_3_(PO_4_)_2_, was formed after pH > 7. For the Ca-Mg-P system, the suitable pH for phosphorus removal was after 5.

**Figure 7:**
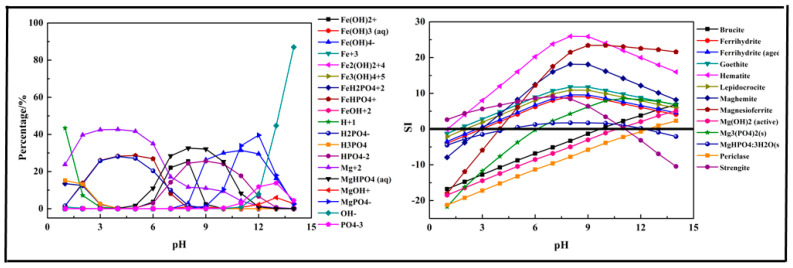
Percentage of species in the Fe-Mg-P-H_2_O system at 298 K at ranged pH ((P) = 0.01 mol/L, (Fe^3+^) = 0.003 mol/L, (Mg^2+^) = 0.0075 mol/L).

The results shown in Figure 7 indicate that the main species in the Fe-Mg-P-H_2_O system could be divided into three sections. At pH = 1–6, H_2_PO_4_^−^, FeHPO_4_^+^, and Mg^2+^ were the main species in the solution. As pH value increased, the above ions transformed into MgHPO_4_ (aq) (pH = 6–11), HPO_4_^2−^ (pH = 6–13), Fe(OH)_4_^−^ (pH > 8), and MgPO_4_^−^ (pH > 9). Strengite was the first predicted precipitation product generated in the precipitation process, and it dissolved again after pH > 11. Mg_3_(PO_4_)_2,_ MgHPO_4_·3H_2_O generated after pH = 6. MgHPO_4_·3H_2_O was formed at pH = 5 and then dissolved after pH > 12.

#### 2.2.3. Fe-Ca-Mg-P-H_2_O System

**Figure 8:**
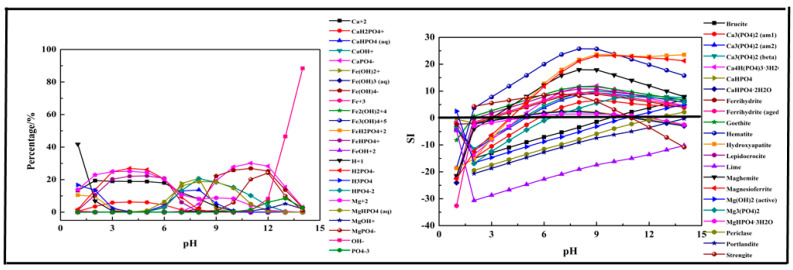
Percentage of species in the Fe-Ca-Mg-P-H_2_O system at 298 K at ranged pH ((P) = 0.01 mol/L, (Fe^3+^) = 0.004 mol/L, (Ca^2+^) = 0.0045 mol/L, (Mg^2+^) = 0.0045 mol/L).

The results shown in Figure 8 indicate that the main species in the Fe-Ca-Mg-P system could be divided into three sections. At pH = 1–6, CaH_2_PO_4_^+^, H_2_PO_4_^−^, Mg^2+^, FeHPO_4_^+^, and Ca^2+^ were the main species in the solution. As pH value increased, above ions transformed into MgHPO_4_ (aq) (pH = 5–13), CaHPO_4_ (aq) (pH = 5–10), HPO_4_^2−^ (5–13), CaPO_4_^−^ (pH = 8–14), Fe(OH)^2+^ (pH = 5–12), and MgPO_4_^−^ (pH = 9–14). The first precipitation containing phosphorus was Strengite after pH > 2, while it occurred at pH = 0 in a single-salt system and two-salts system. Then other precipitations formed continually. The composition in the three salts system was more complex, and the precipitation was no more stable than the single salt system.

### 2.3. Phosphorous Removal Experiments

**Figure 9:**
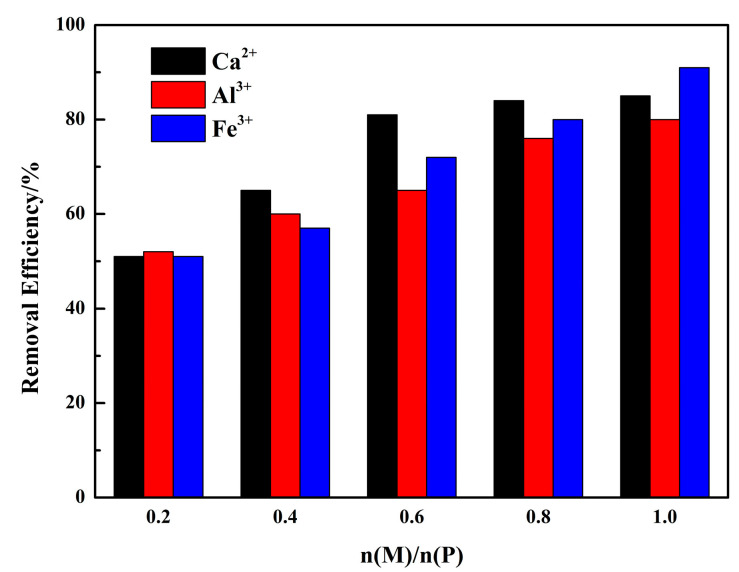
Phosphorous removal experiments ((P) = 0.01 mol/L, n(Ca)/n(P) = 0.2–1, n(Al)/n(P) = 0.2–1, n(Fe)/n(P) = 0.2–1).

The phosphorous removal experiments were conducted with Ca^2+^, Al^3+^, and Fe^3+^. The results are shown in Figure 9. The phosphorus ion was precipitated under the selected reaction conditions. The dosage of metal ions had a significant effect on the removal efficiency of phosphorous. For Ca^2+^ salts, the high removal efficiency was 81.25% and achieved at n(Ca^2+^)/n(P) = 0.6. A further increase in Ca had no obvious effect on the removal efficiency. While for Al^3+^ salts, the removal efficiency was a step increase, and the highest was 80.13%. Great removal performance was achieved by Fe^3+^ at n(Fe)/n(P) = 1 with a removal efficiency of 91.54%. These actual experimental results are consistent with the analysis above, indicating that the theoretical analysis could be used to guide the actual experiments.

## 3. Materials and Methods

The thermodynamic equilibrium diagrams of P-H_2_O system and P-M-H_2_O system (M stands for Fe, Al, Ca, Mg) were simulated by the VISUAL MINTEQ software. The concentration of P and M are detailed in Table 1. For the P-H_2_O system, the concentration of phosphorus ranged from 0.01 mol/L to 0.09 mol/L. In the Fe-P-H_2_O system, the concentration was (P) = 0.01 mol/L, (Fe^3+^) = 0.01 mol/L. For theCa-P-H_2_O system, the concentration was (P) = 0.01 mol/L, (Ca^2+^) = 0.015 mol/L. For the Mg-P-H_2_O system, the concentration was (P) = 0.01 mol/L, (Mg^2+^) = 0.015 mol/L. For the Fe-Mg-P-H_2_O system, the concentration was (P) = 0.01 mol/L, (Fe^3+^) = 0.005 mol/L, (Mg^2+^) = 0.0075 mol/L. For the Ca-Mg-P-H_2_O system, the concentration was (P) = 0.01 mol/L, (Ca^2+^) = 0.0075 mol/L, (Mg^2+^) = 0.0075 mol/L. For the Fe-Ca-P-H_2_O system, the concentration was (P) = 0.01 mol/L, (Fe^3+^) = 0.003 mol/L, (Ca^2+^) = 0.0075 mol/L. For Fe-Ca-Mg-P-H_2_O system, the concentration was (P) = 0.01 mol/L, (Fe^3+^) = 0.004 mol/L, (Ca^2+^) = 0.0045 mol/L, (Mg^2+^) = 0.0045 mol/L. The activity coefficients of the charged materials were calculated with the Davies equation. Meanwhile, the saturation index (SI) was used to predict the trend of precipitated and dissolved species and was calculated based on Equation (1). If SI > 0, the content was in oversaturation and might precipitate; SI = 0, the content was in an equilibrium state; SI < 0, the content existed in the form of ions in the solution.

(4) SI=log IAP − logKs

where IAP is the selected ion activity in the Visual MINTEQ software, and Ks is the solubility product constant.

## 4. Conclusions

Based on the results obtained in this study, the following conclusions can be obtained:

(1) The phosphorus ions existed in the form of H_3_PO_4_, H_2_PO_4_^−^, HPO_4_^2−^, and PO_4_^3−^. Among them, H_2_PO_4_^−^ and HPO_4_^2−^ were the main species in the acidic medium (99% at pH = 5) and alkaline medium (97.9% at pH = 10). In the P-Fe-H_2_O System ((P) = 0.01 mol/L, (Fe^3+^) = 0.01 mol/L), H_2_PO_4_^−^ was transformed to FeHPO_4_^+^ at pH = 0–7 due to the existence of Fe^3+^ and then transformed into HPO_4_^2−^ at pH > 6 as the Fe^3+^ was mostly precipitated. In the P-Ca-H_2_O System ((P) = 0.01 mol/L, (Ca^2+^) = 0.015 mol/L), the main species in the acidic medium were CaH_2_PO_4_^+^ and HPO_4_^2−^, and then transformed into CaPO_4_^−^ at pH > 7. In the P-Mg-H_2_O System ((P) = 0.01 mol/L, (Mg^3+^) = 0.015 mol/L), the main species in the acidic medium was H_2_PO_4_^−^ and then transformed into MgHPO_4_ at pH = 5–10, and finally transformed into MgPO_4_^−^ as pH increased.
(2) The phosphorus was more easily precipitated in the P-Fe-H_2_O system than the P-Ca-H_2_O system and P-Mg-H_2_O system. The suitable pH of the solution for phosphorus precipitation was about 5–10 in all precipitation systems.
(3) The verification experiments (precipitation experiments) with single metal ions confirm that the theoretical analysis can be used to guide the actual experiments.

## Figures and Tables

**Table 1:** The concentration for simulating process.

System	Concentration (mol/L)			
	P	Fe	Ca	Mg
P-H_2_O	0.01–0.09			
Fe-P-H_2_O	0.01	0.01		
Ca-P-H_2_O	0.01		0.015	
Mg-P-H_2_O	0.01			0.015
Fe-Mg-P-H_2_O	0.01	0.005		0.0075
Ca-Mg-P-H_2_O	0.01		0.0075	0.0075
Fe-Ca-P-H_2_O	0.01	0.005	0.0075	
Fe-Ca-Mg-P-H_2_O	0.01	0.004	0.0045	0.0045

## Data Availability

No new data were created or analyzed in this study. Data sharing is not applicable to this article

## Sample Availability

Not available.
